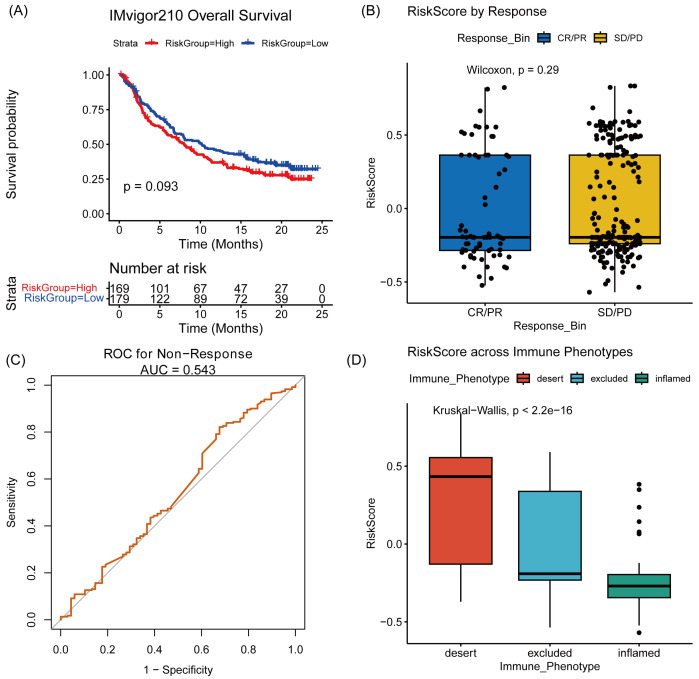# Vasoactive intestinal peptide associated 8-gene signature with prognostic and immune associations in melanoma

**DOI:** 10.1097/MD.0000000000048961

**Published:** 2026-05-29

**Authors:** Xiaogang Hong, Xiuzhen Zheng

**Affiliations:** a Department of Ophthalmology, People’s Hospital of Kaihua, Quzhou, Zhejiang, China.

**Keywords:** immune cell infiltration, machine learning, melanoma, nomogram, vasoactive intestinal peptide

## Abstract

Vasoactive intestinal peptide (VIP) plays a multifaceted role in cancer biology, yet its prognostic and immunological implications in melanoma remain underexplored. This study aimed to construct a VIP-related gene signature for risk stratification and to explore its potential associations with the tumor immune microenvironment and drug sensitivity in melanoma. RNA-sequencing data and clinical information were obtained from TCGA-SKCM (n = 477, training set) and GSE65904 (n = 209, validation set). A VIP-related prognostic signature (VIPsig) was developed using stepwise Cox regression and Gradient Boosting Machine algorithms. Patients were stratified into high- and low-risk groups based on the median risk score. Somatic mutations, immune infiltration, drug sensitivity, and functional enrichment were further analyzed. A nomogram integrating clinical factors was developed and validated. Single-cell RNA-seq data (GSE115978) were reanalyzed to characterize cellular expression patterns. A preliminary pan-cancer analysis indicated potential dysregulation of VIP pathway genes. In melanoma, we identified an 8-gene signature (*CD28*, *CD80*, *CD86*, *CTLA4*, *FAS*, *IFNG*, *IL10*, and *IL12A*) associated with survival. In the training set, high-risk patients exhibited significantly worse overall survival (OS) compared to low-risk patients (HR = 2.96, 95% CI: 2.24–3.92, *P* < .001). This was validated in the GSE65904 cohort (HR = 1.87, 95% CI: 1.26–2.77, *P* = .002). The VIPsig demonstrated discriminative ability with 1-, 3-, and 5-year AUCs of 0.760 (95% CI: 0.675–0.845, *P* < .001), 0.749 (95% CI: 0.692–0.807, *P* < .001), and 0.770 (95% CI: 0.717–0.823, *P* < .001) in the training set, respectively. Functionally, the signature was significantly associated with immune cell infiltration and immune checkpoint expression. In silico drug sensitivity analysis suggested potential associations with estimated IC50 values for several agents, though these findings require experimental validation. A nomogram integrating VIPsig with clinicopathological factors showed improved net benefit in decision curve analysis. The VIP-based signature demonstrates robust prognostic value for melanoma survival and reflects the tumor immune microenvironment status. Exploratory analyses suggest potential associations with in silico drug sensitivity estimates; however, its utility for treatment response prediction remains unvalidated and warrants further investigation in prospective melanoma cohorts treated with immune checkpoint inhibitors.

## 1. Introduction

Cutaneous melanoma is an aggressive malignancy originating from melanocytes, with a steadily rising global incidence over the past decades.^[1,2]^ It accounts for the majority of skin cancer-related deaths due to its high metastatic potential and resistance to conventional therapies.^[3,4]^ Current estimates indicate over 3,00,000 new cases are diagnosed worldwide annually, representing a significant public health burden.^[1]^ Although immune checkpoint inhibitors (ICIs) have revolutionized melanoma treatment,^[5,6]^ a significant subset of patients exhibits intrinsic or acquired resistance.^[7,8]^ Several gene signatures have been developed to predict prognosis and treatment outcomes, focusing on tumor mutation burden, T-cell exclusion, or inflammatory cytokine profiles.^[9-11]^ While these models have significantly advanced the field, they primarily capture general characteristics of the tumor immune microenvironment (TIME) and often overlook the neuroimmunological axis.^[12]^ The crosstalk between the nervous and immune systems, particularly through neuropeptides, has emerged as a critical but underexplored regulator of tumor progression and immune tolerance. Therefore, existing signatures may lack the specificity to capture immune evasion mechanisms driven by neuroendocrine signaling, representing a critical knowledge gap.

The vasoactive intestinal peptide (VIP) is a multifunctional neuropeptide that exerts potent immunomodulatory effects through binding to its receptors, *VPAC1* and *VPAC2*. Beyond its physiological roles, VIP signaling has been implicated in the pathogenesis of various cancers. It can directly influence tumor cell proliferation, migration, and survival in contexts such as breast, prostate, and colorectal cancer.^[13-16]^ More notably, VIP is a pivotal regulator of immune homeostasis within the tumor microenvironment (TME).^[17]^ It promotes a shift towards an anti-inflammatory phenotype by stimulating regulatory T-cell (Treg) differentiation, inhibiting pro-inflammatory cytokine production (e.g., TNF-α, IL-6), and alternatively activating macrophages.^[18-20]^ This immunosuppressive activity suggests a role for VIP in facilitating tumor immune evasion. However, its specific function and clinical relevance in melanoma – a malignancy profoundly shaped by the TIME – are complex and not fully elucidated.^[21]^ Emerging evidence suggests that VIP and its related genes may influence melanoma progression and response to therapy,^[22]^ positioning them as potential biomarkers and therapeutic targets worthy of systematic investigation.

Based on this biological premise, we hypothesized that the dysregulation of VIP-related genes constitutes a distinct “neuro-immune” signature that correlates with aggressive melanoma phenotypes and immune suppression, offering prognostic information nonredundant to existing markers. To test this hypothesis, this study addressed 3 prespecified objectives: to construct a robust VIP-based gene signature (VIPsig) using machine learning algorithms in bulk transcriptomic cohorts; to validate the prognostic accuracy of VIPsig and its association with the immune landscape; and to perform an exploratory analysis of its associations with drug sensitivity. It is important to note that this study utilizes retrospective multi-cohort data to establish prognostic associations, and the findings regarding therapeutic sensitivity are hypothesis-generating, warranting further validation in prospective clinical trials.

## 2. Materials and methods

### 2.1. Data acquisition and processing

RNA-sequencing data (FPKM values) for skin cutaneous melanoma (SKCM, n = 477) were obtained from The Cancer Genome Atlas (TCGA, https://portal.gdc.cancer.gov/) repository and used as the training cohort. The validation cohort GSE65904 (n = 209) was obtained from the Gene Expression Omnibus (https://www.ncbi.nlm.nih.gov/geo/). Each dataset underwent rigorous filtering to exclude samples with incomplete clinical or survival information. Batch effect correction was performed by merging the 2 datasets and applying the ComBat algorithm (sva package v3.56.0; Boston University) to the combined expression matrix, as detailed in the uploaded analysis code. The corrected matrix was then split back into TCGA-SKCM and GSE65904 portions. Importantly, all parameters for VIPsig construction (gene filtering, StepCox[forward] selection, and Gradient Boosting Machine [GBM] coefficient estimation) were derived exclusively from the TCGA-SKCM training set. The GSE65904 cohort was used to evaluate the performance of the generated candidate models, thereby informing the comparison of machine learning algorithms and the final selection of the optimal prognostic signature. Thirteen VIP-related genes from the WP_CONTROL_OF_IMMUNE_TOLERANCE_BY_VASOACTIVE_INTESTINAL_PEPTIDE gene set were retrieved from the MSigDB (https://www.gsea-msigdb.org/gsea/msigdb) database (Table S1, Supplemental Digital Content).^[23]^ The CONSORT diagram was illustrated in Figure S1, Supplemental Digital Content.

### 2.2. Pan-cancer analysis

The TCGAplot package (version 8.0.0; Huazhong University of Science and Technology) was employed for pan-cancer analysis.^[24]^ Gene set variation analysis was used to calculate enrichment scores for the VIP gene set. Wilcoxon tests were applied to compare scores between tumor and normal tissues. Spearman’s correlation analysis was conducted to evaluate associations between the VIP gene set score and tumor mutational burden (TMB) or microsatellite instability (MSI). Cox proportional hazards regression was used to assess the relationship between the VIP score and overall survival across cancer types, visualized via forest plots.

### 2.3. Machine learning model construction

The Mime1 package (version 0.0.0.9000; Central South University)^[25]^ was used to develop and evaluate machine learning-based integrative models from transcriptomic data. Model construction and performance evaluation were conducted using the ML.Dev.Prog.Sig() function within a 10-fold cross-validation framework. Regarding parameter configuration, nodesize – a critical hyperparameter for the random survival forest model was set to 5, and unicox.filter.for.candi was set to TRUE, while all other parameters retained their default values. The procedure initially identified VIP-related genes significantly associated with overall survival (OS) in the TCGA-SKCM cohort. Subsequently, based on the expression profiles of these candidate genes, 117 combinatorial models were established by integrating 10 machine learning algorithms: random survival forest, Enet, StepCox, CoxBoost, plsRcox, superpc, GBM, survivalsvm, Ridge, and Lasso. Finally, the prognostic risk score, designated as VIPsig, was calculated for each model. Following the standard model selection procedure recommended by the Mime framework,^[26]^ the optimal model was selected based on the highest mean concordance index (C-index) across the validation cohort(s). This approach prioritizes generalizability and reduces the risk of overfitting by evaluating model performance on data not used for parameter estimation, consistent with established practices in machine learning-based prognostic model development.^[26-28]^ Importantly, while all model parameters (gene coefficients and weights) were estimated exclusively using the TCGA-SKCM cohort, the GSE65904 cohort was used as an integral part of the selection process to identify the algorithm with the highest generalizability. Samples in both TCGA-SKCM and GSE65904 cohorts were stratified into high- and low-risk groups based on the median value of the VIPsig. Kaplan–Meier survival analysis was used to compare overall survival between risk groups. Time-dependent receiver operating characteristic (ROC) curves were constructed to evaluate discriminative performance at 1, 3, and 5 years. The area under the curve (AUC), its 95% confidence interval, and the associated *P*-value were computed using the DeLong nonparametric method as implemented in the pROC R package (version 1.18.5; University of Geneva).^[29]^ The *P*-value tests the null hypothesis H_0_: AUC = 0.5 (no discriminative ability) against the alternative H_1_: AUC ≠ 0.5. Spearman’s rank correlation was used for all correlation analyses throughout this study, as the variables assessed (risk scores, immune infiltration estimates, IC50 values, and genomic instability metrics) do not assume normal distribution. All correlation coefficients reported in figures and text refer to Spearman’s ρ unless explicitly stated otherwise. The IMvigor210 cohort (metastatic urothelial carcinoma treated with anti-PD-L1 agent atezolizumab) was analyzed using the IMvigor210CoreBiologies package (version 1.0.1). The association between VIPsig and objective response to immunotherapy (complete/partial response vs stable/progressive disease) was assessed using the Wilcoxon rank-sum test and ROC analysis.

### 2.4. Somatic mutation analysis

Somatic mutation data were analyzed using the R package maftools (v2.24.0; National University of Singapore).^[30]^ Mutational signatures were extracted via Non-Negative Matrix Factorization and matched against the COSMIC legacy database to quantify UV exposure (Signature 7). Tumor Mutational Burden (TMB) was calculated as non-synonymous mutations per megabase. To assess the association between the Risk Score and TMB, partial Spearman’s correlation analysis was performed using the ppcor (version 1.1; Wayne State University) package, adjusting for tumor stage, sample type, and UV signature contribution. Additionally, multivariable logistic regression was employed to evaluate the independent association between risk groups and the top 20 mutated genes, correcting for the aforementioned covariates. Results were reported as odds ratios with Benjamini–Hochberg adjusted *P*-values.

### 2.5. Immune landscape and drug sensitivity analysis

The IOBR package (version 0.99.0; Southern Medical University) was used to evaluate the immune landscape.^[31]^ CIBERSORT estimated infiltration levels of 22 immune cell types. Immunophenoscore (IPS) was calculated using the IPS algorithm, and stromal/immune/ESTIMATE scores along with tumor purity were computed via the ESTIMATE algorithm. Group differences were assessed using Wilcoxon tests, and Spearman’s correlation analyzed associations between VIPsig (and its component genes) and IPS, ESTIMATE scores, and immune checkpoint gene expression. Following the assessment of the immune landscape, we extended our analysis to explore potential therapeutic vulnerabilities associated with the VIPsig using the pRRophetic R package (version 0.5; University of Chicago).^[32]^ This algorithm employs ridge regression models trained on the Genomics of Drug Sensitivity in Cancer database, which encompasses extensive pharmacogenomic data from a wide panel of cancer cell lines. We estimated the half-maximal inhibitory concentration (IC50) for each sample – where a lower IC50 indicates higher predicted sensitivity Group differences in drug sensitivity were compared via Wilcoxon test.

### 2.6. Differential expression and enrichment analysis

The limma package (version 3.64.3; WEHI) was used to identify differentially expressed genes (DEGs) between high- and low-risk groups, with thresholds of |log_2_(fold change)| > 1 and adjusted *P*-value < .05.^[33]^ Gene Ontology (GO) and Kyoto Encyclopedia of Genes and Genomes (KEGG) pathway enrichment analyses were performed on DEGs using clusterProfiler package (version 4.16.0; Southern Medical University).^[34]^ Gene Set Enrichment Analysis was conducted using the Hallmark gene sets (h.all.v2025.1.Hs.symbols.gmt) from MSigDB to identify coordinated phenotypic changes.

### 2.7. Nomogram construction

The rms (version 8.1-0; Vanderbilt University) and survival (version 3.8-3; Mayo Clinic) packages were used to develop and validate a clinical prediction model. The model integrated VIPsig and clinicopathological features (age, sex, pT stage, pN stage, pM stage, pathologic stage, Clark level, and radiotherapy). Univariate Cox regression (*P* < .05) identified variables associated with OS, which were included in a multivariate Cox model to determine independent prognostic factors. For Cox regression analyses and nomogram construction, staging and grading variables were coded as ordered numeric integers as follows: pT stage (T1 = 1, T2 = 2, T3 = 3, T4 = 4), pN stage (N0 = 0, N1 = 1, N2 = 2, N3 = 3), pM stage (M0 = 0, M1 = 1), pathologic stage (Stage I = 1, Stage II = 2, Stage III = 3, Stage IV = 4), and Clark level (Level I = 1, Level II = 2, Level III = 3, Level IV = 4, Level V = 5). This ordered numeric coding approach assumes a monotonic relationship between increasing stage/grade and survival risk. Age and VIPsig risk score were included as continuous variables. A nomogram was constructed using the cph() and nomogram() functions to predict 1‑, 3‑, and 5‑year OS. Internal validation was performed via bootstrap resampling (1000 repetitions). Calibration curves and decision curve analysis assessed predictive accuracy and clinical utility, respectively. Time-dependent ROC curves evaluated discrimination ability at 1, 3, and 5 years.

### 2.8. Single-cell RNA sequencing analysis

The scRNA-seq dataset (GSE115978) was processed using the Seurat R package (version 5.4.0; New York Genome Center).^[35]^ Raw count matrices were filtered to exclude genes expressed in fewer than 3 cells and cells with fewer than 200 detected features. Gene expression was normalized using the global-scaling LogNormalize method (scale factor = 10,000), followed by the identification of the top 2000 variable features via variance-stabilizing transformation. Principal component analysis was performed on scaled data, and the first 15 principal components were selected for nonlinear dimensionality reduction using t-distributed Stochastic Neighbor Embedding (t-SNE). Cell annotations were derived from the original study’s metadata and rigorously validated by visualizing the expression of canonical lineage markers: *PMEL*/*MLANA* (Melanoma), *CD8A*/*CD3D* (CD8+ T cells), *CD4*/*IL7R* (CD4+ T cells), *GNLY*/*NKG7* (NK cells), *MS4A1* (B cells), *CD14* (Myeloid), *PECAM1* (Endothelial), and *COL1A1* (Cancer-associated fibroblasts). To quantify the risk signature at single-cell resolution, a composite Risk Score was calculated for each cell using the AddModuleScore function based on the model-derived gene signature. Statistical differences in risk scores between treatment groups (Naive vs Treated) within malignant subpopulations were assessed using the Wilcoxon rank-sum test.

## 3. Results

### 3.1. Pan-cancer analysis of VIP-related gene sets

**Figure 1.**
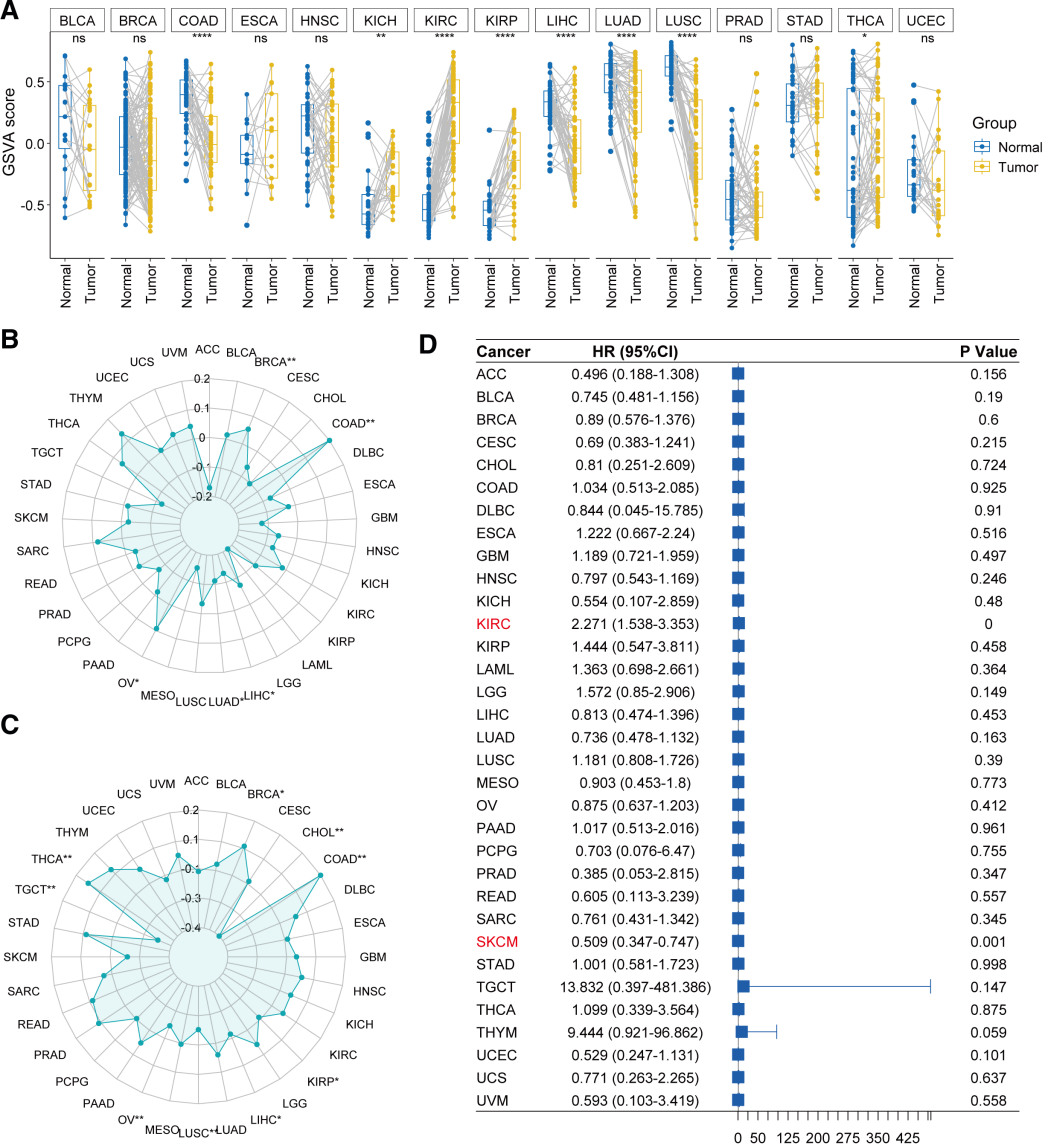
Pan-cancer analysis of GSVA scores for the VIP gene set. (A) Wilcoxon test comparing VIP scores between tumor and normal tissues. Spearman’s rank correlation analysis between VIP score and (B) TMB, and (C) MSI. (D) Forest plot of Cox analysis evaluating the association between VIP score and overall survival across cancers. *P < .05, **P < .01, ****P < .0001. GSVA = Gene Set Variation Analysis, MSI = microsatellite instability, Ns = not significant, TMB = tumor mutational burden, VIP = vasoactive intestinal peptide.

The enrichment score of the VIP gene set was calculated using the Gene set variation analysis algorithm. The results revealed that this score was dysregulated across multiple cancer types, showing significant upregulation or downregulation. Compared to normal tissues, the score was significantly decreased in COAD, LIHC, LUAD, and LUSC, while it was markedly increased in KICH, KIRC, KIRP, and THCA (Fig. 1A). Furthermore, correlation analysis demonstrated that the VIP score was significantly associated with TMB in BRCA, COAD, LIHC, LUAD, and OV (Fig. 1B), and with MSI in BRCA, CHOL, COAD, KIRP, LIHC, LUSC, OV, TGCT, and THCA (Fig. 1C). Cox analysis indicated that the VIP score was significantly correlated with overall survival in KIRC (HR = 2.271, 95% CI: 1.538–3.353, *P* < .001) and SKCM (HR = 0.509, 95% CI: 0.347–0.747; Fig. 1D). These findings suggest that the VIP gene set is involved in tumorigenesis and progression across multiple cancer types, exhibiting pleiotropic effects.

### 3.2. Construction and evaluation of the VIPsig risk signature

**Figure 2.**
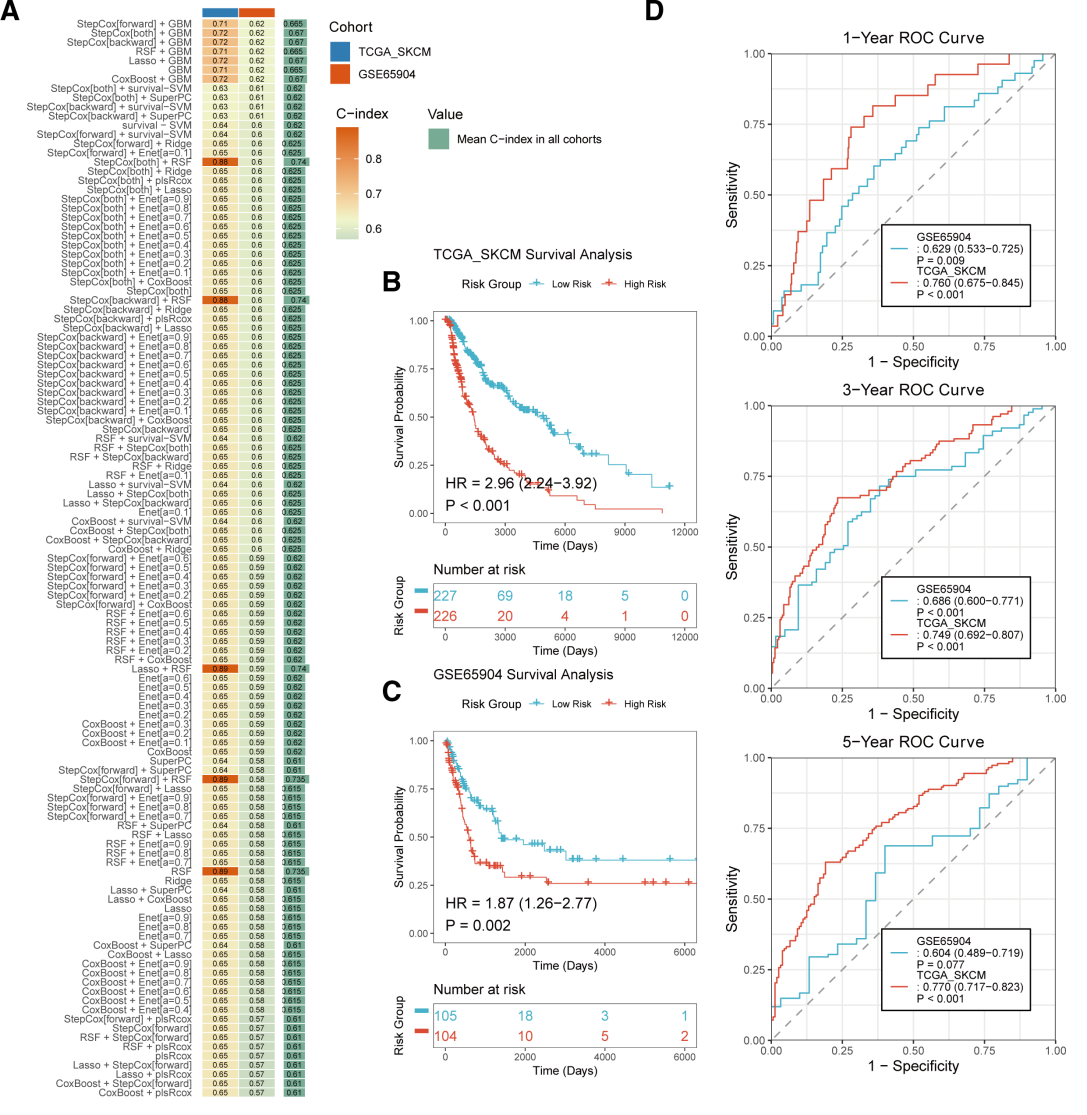
Construction of the VIPsig risk signature based on VIP-related genes in melanoma. (A) Distribution of C-index values across 117 machine learning-based signature models. Kaplan–Meier survival analysis comparing overall survival between high- and low-risk groups in the (B) TCGA-SKCM and (C) GSE65904 cohorts. (D) ROC curves showing the area under the curve (AUC) of VIPsig for predicting 1-, 3-, and 5-year overall survival. ROC = receiver operating characteristic, VIP = vasoactive intestinal peptide.

Cox analysis identified 8 genes significantly associated with overall survival in melanoma: *CD28*, *CD80*, *CD86*, *CTLA4*, *FAS*, *IFNG*, *IL10*, and *IL12A*. Subsequently, 117 machine learning models were constructed based on these genes (Fig. 2A). Among 117 machine learning models, the StepCox[forward] + GBM algorithm achieved the highest mean C-index across the validation cohort and was therefore selected as the optimal model, following the Mime framework’s recommended selection criterion.^[26]^ The performance of the top 10 candidate models in both training and validation cohorts is detailed in Table S2, Suppplemental Digital Content, confirming consistent performance of the selected model across datasets. This optimal model was designated as VIPsig. Kaplan–Meier survival analysis demonstrated that patients in the high-risk group (n = 227) had significantly worse OS compared to the low-risk group (n = 226) in the training cohort (HR = 2.96, 95% CI: 2.24–3.92, *P* < .001; Fig. 2B). This finding was validated in the external GSE65904 cohort (HR = 1.87, 95% CI: 1.26–2.77, *P* = .002; Fig. 2C). ROC curve analysis indicated robust predictive performance (Fig. 2D). In the TCGA-SKCM cohort, the AUCs for 1-, 3-, and 5-year OS were 0.760 (95% CI: 0.675–0.845, *P* < .001 vs null AUC = 0.5, DeLong test), 0.749 (95% CI: 0.692–0.807, *P* < .001, DeLong test), and 0.770 (95% CI: 0.717–0.823, *P* < .001, DeLong test), respectively. In the GSE65904 cohort, the AUCs for 1-, 3-, and 5-year OS were 0.629 (95% CI: 0.533–0.725, *P* = .009, DeLong test), 0.686 (95% CI: 0.600–0.771, *P* < .001, DeLong test), and 0.604 (95% CI: 0.489–0.719, *P* = .077, DeLong test), respectively.

To rigorously validate the robustness and broad applicability of VIPsig, we performed a series of prespecified sensitivity and subgroup analyses. As illustrated in the forest plot (Fig. S2A, Supplemental Digital Content), the prognostic power of VIPsig remained highly consistent and statistically significant across strictly defined sensitivity cohorts. Furthermore, to verify the diagnostic stability across diverse clinical scenarios, we conducted stratified time-dependent ROC analyses (Fig. S2B, Supplemental Digital Content). The VIPsig demonstrated stable predictive accuracy (1-, 3-, and 5-year AUCs) across distinct patient strata, including gender, Clark level, sample type, and pathological stages (Stage I + II vs III + IV), confirming its reliability independent of confounding clinical variables.

### 3.3. Association between VIPsig and clinicopathological features in melanoma patients

**Figure 3.**
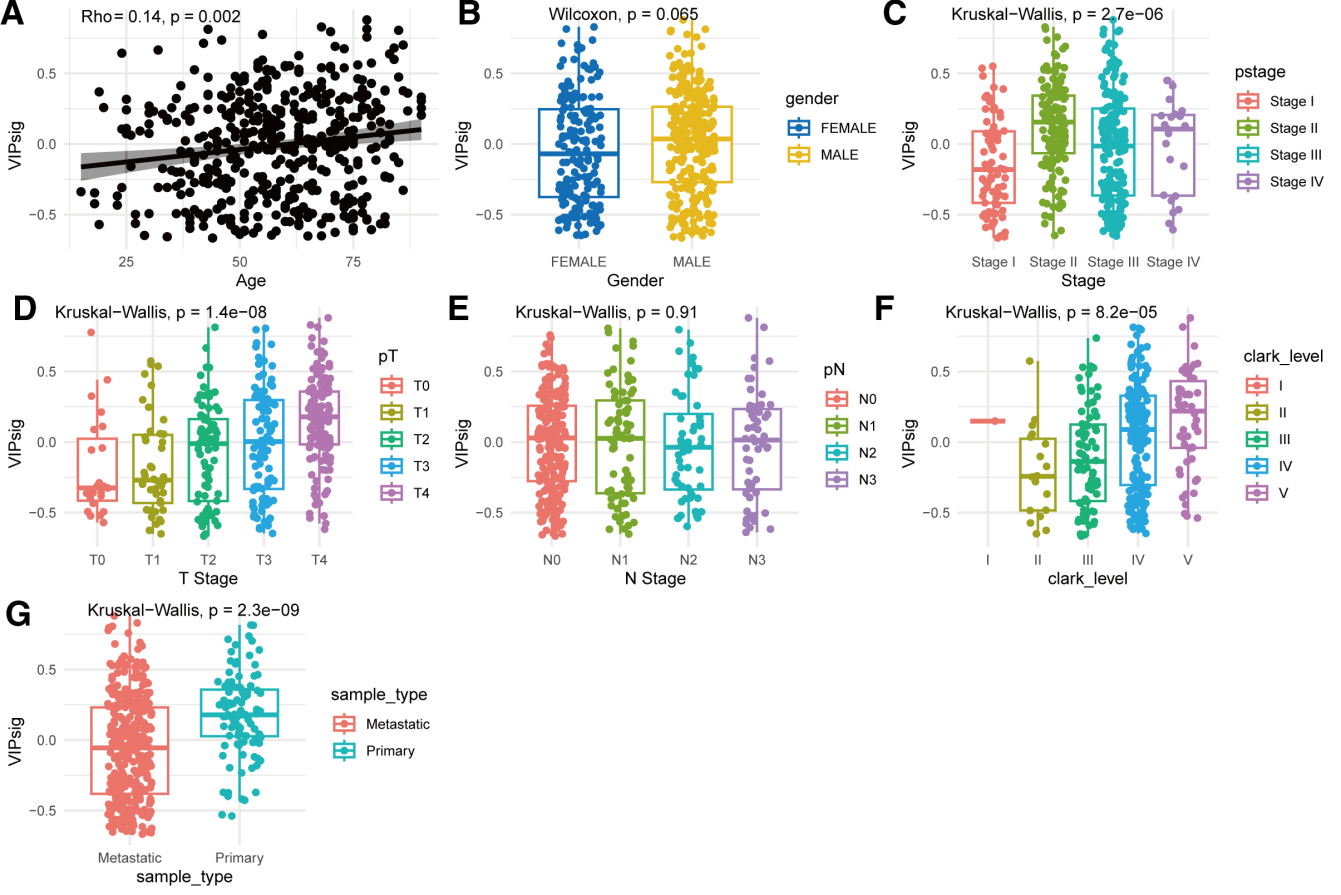
Spearman’s rank correlation between VIPsig and clinicopathological features in melanoma. (A) Scatter plot of Spearman correlation analysis between patient age and VIPsig. Kruskal–Wallis test comparing VIPsig across subgroups of (B) gender, (C) pathological stage, (D) T stage, (E) N stage, (F) Clark level, and (G) sample type. VIP = vasoactive intestinal peptide.

We next investigated the relationship between VIPsig and clinicopathological characteristics of melanoma patients. A significant positive correlation was observed between VIPsig and patient age (ρ = 0.14, *P* = .002; Fig. 3A). Subgroup analysis based on clinicopathological features revealed no significant difference in VIPsig between gender subgroups (Fig. 3B), whereas significant differences were detected across pathological stages (*P* = 2.7e−06; Fig. 3C), T stages (*P* = 1.4e−08; Fig. 3D), and Clark levels (*P* = 8.2e−05; Fig. 3F). No significant difference was observed across N stages (Fig. 3E). Additionally, metastatic melanoma patients exhibited significantly lower VIPsig scores compared to those with primary tumors (*P* = 2.3e−09; Fig. 3G). These results indicate that VIPsig is associated with age and pathological features in melanoma patients.

### 3.4. VIPsig and the immune landscape of melanoma

**Figure 4.**
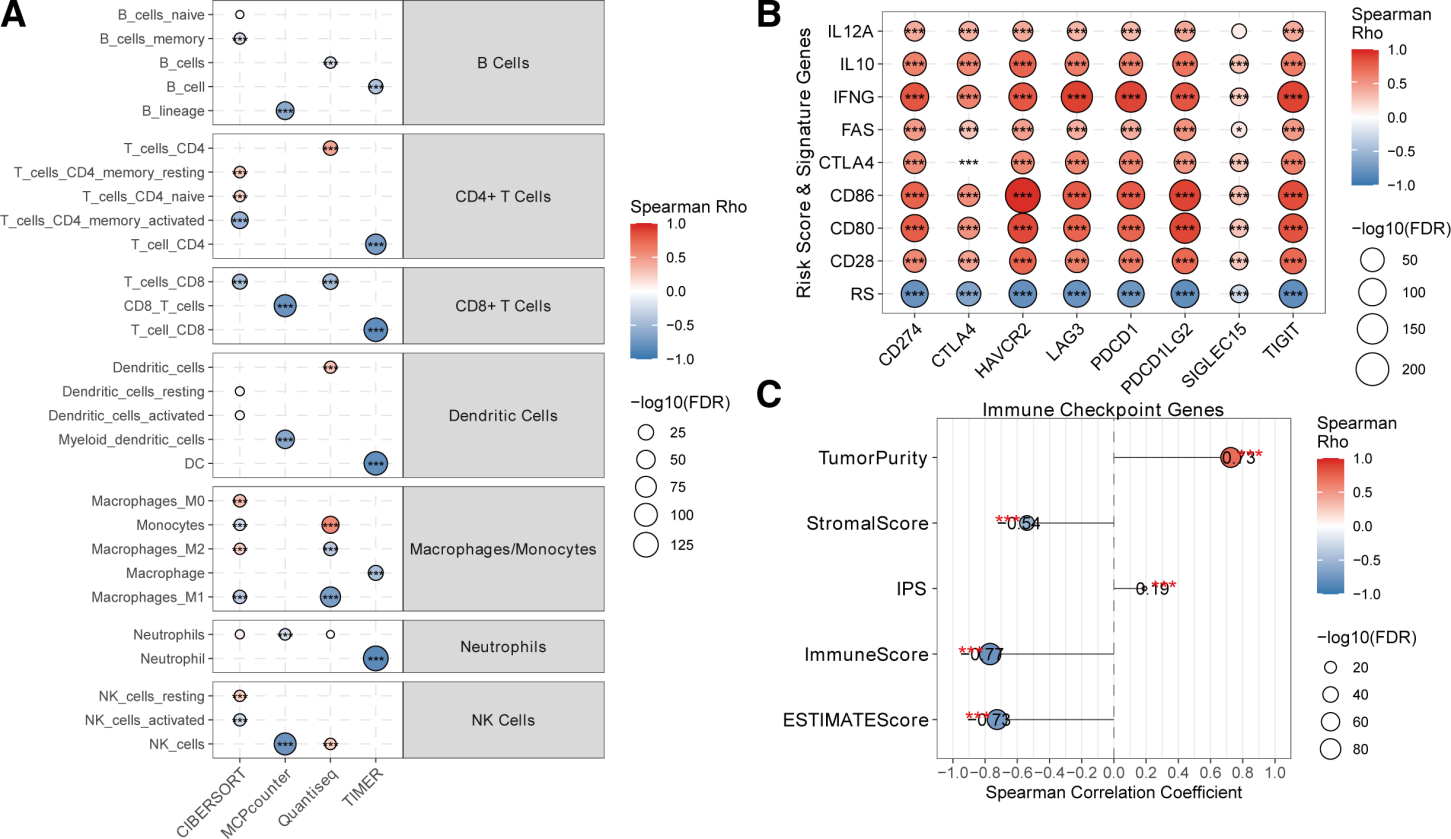
The immune landscape of the VIPsig risk signature. (A) Cross-validation of immune cell infiltration correlations using 4 independent algorithms (CIBERSORT, MCPcounter, Quantiseq, and TIMER). (B) Dot plot illustrating the correlation between the VIPsig Risk Score (and its constituent genes) and key immune checkpoint genes (ICGs). Asterisks denote FDR-adjusted significance levels (*FDR < 0.05, **FDR < 0.01, ***FDR < 0.001). (C) Lollipop chart showing the correlation of the Risk Score with Tumor Purity, Stromal Score, Immune Score, ESTIMATE Score, and immunophenoscore (IPS). ICG = immune checkpoint gene, FDR = false discovery rate, VIP = vasoactive intestinal peptide.

To robustly characterize the immune contexture associated with VIPsig, we performed a cross-platform deconvolution analysis using CIBERSORT, MCP-counter, Quantiseq, and TIMER. Despite methodological differences, consistent patterns emerged: the VIPsig risk score was significantly negatively correlated with the infiltration of antitumor effector cells, specifically CD8+ T cells and activated NK cells, across multiple algorithms (Fig. 4A). Conversely, a positive correlation was observed with pro-tumorigenic cell types, such as M0 and M2 macrophages. Furthermore, the VIPsig risk score exhibited a strong negative correlation with the expression of all analyzed immune checkpoint genes (e.g., *CD274*, *CTLA4*, *PDCD1*), whereas its constituent genes showed the opposite trend (Fig. 4B). Consistent with an “immune-cold” phenotype, VIPsig was significantly positively correlated with Tumor Purity (ρ = 0.73) and negatively correlated with Immune Score (ρ = −0.77), Stromal Score (ρ = −0.54), and ESTIMATE Score (ρ = −0.73; Fig. 4C). Interestingly, a weak positive correlation was observed with IPS (ρ = 0.19).

### 3.5. VIPsig and drug sensitivity in melanoma

**Figure 5.**
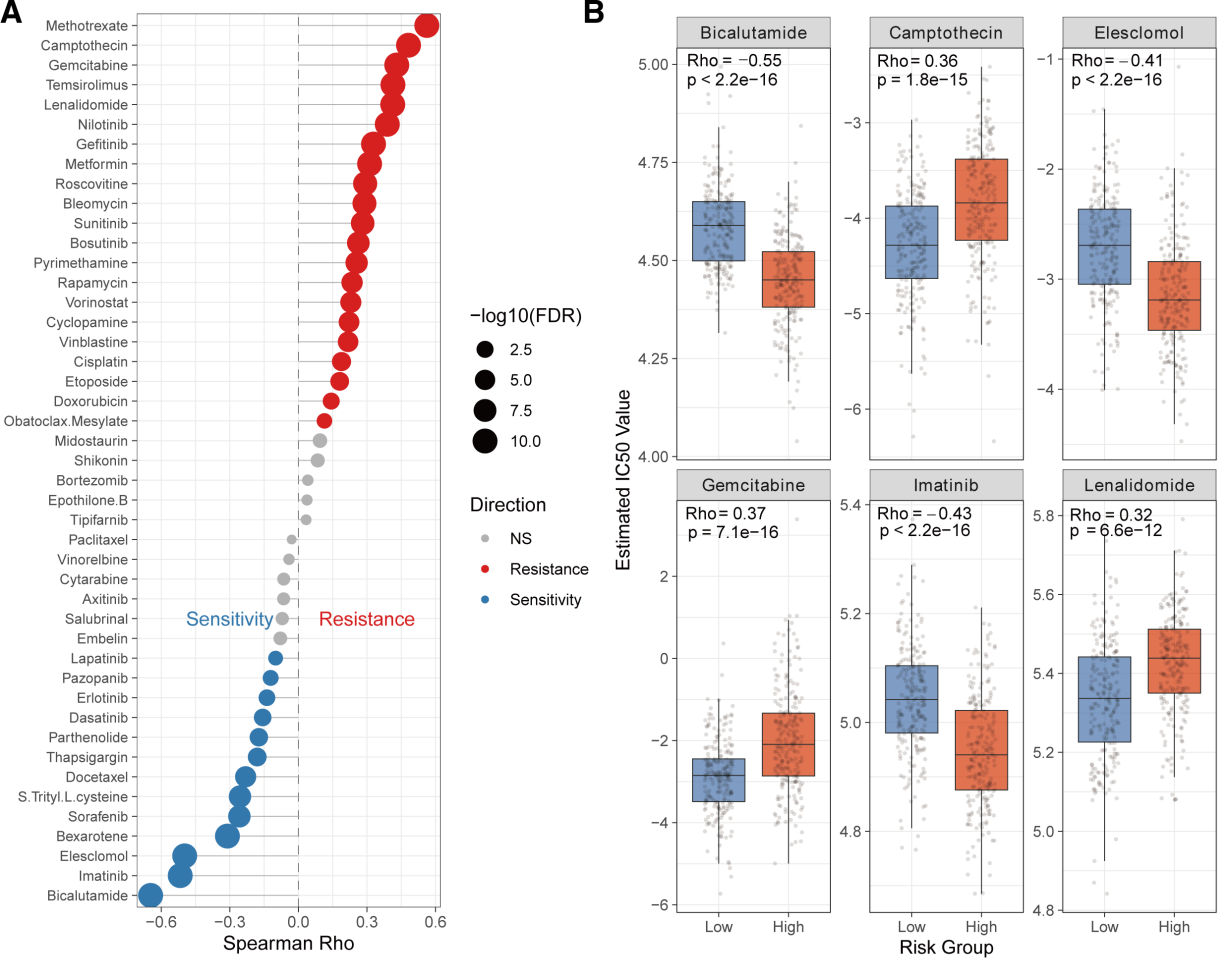
Drug sensitivity and resistance prediction. (A) Lollipop chart displaying the Spearman correlation between the VIPsig Risk Score and the predicted IC50 values of therapeutic agents. (B) Boxplots validating the IC50 differences for the top correlated drugs between low- and high-risk groups. VIP = vasoactive intestinal peptide.

We further performed an exploratory in silico analysis to examine the association between VIPsig and predicted drug sensitivity profiles. To ensure statistical reliability, all associations were corrected for multiple testing (false discovery rate [FDR]). As shown in Figure 5A, VIPsig was positively correlated with the estimated IC50 values of 21 drugs (ρ > 0, red), indicating potential resistance in high-risk patients to agents such as Methotrexate, Camptothecin, and Gemcitabine. Conversely, VIPsig was negatively correlated with the IC50 values of 13 drugs (ρ < 0, blue), suggesting that high-risk patients might be more sensitive to targeted therapies including Bicalutamide, Imatinib, and Elesclomol. These computational predictions were further corroborated by comparing IC50 distributions, where the high-risk group demonstrated significantly lower IC50 values for Bicalutamide (ρ = −0.55, *P* < 2.2e−16) and Imatinib (ρ = −0.43, *P* < 2.2e−16) compared to the low-risk group (Fig. 5B).

### 3.6. VIPsig and gene expression in melanoma

**Figure 6.**
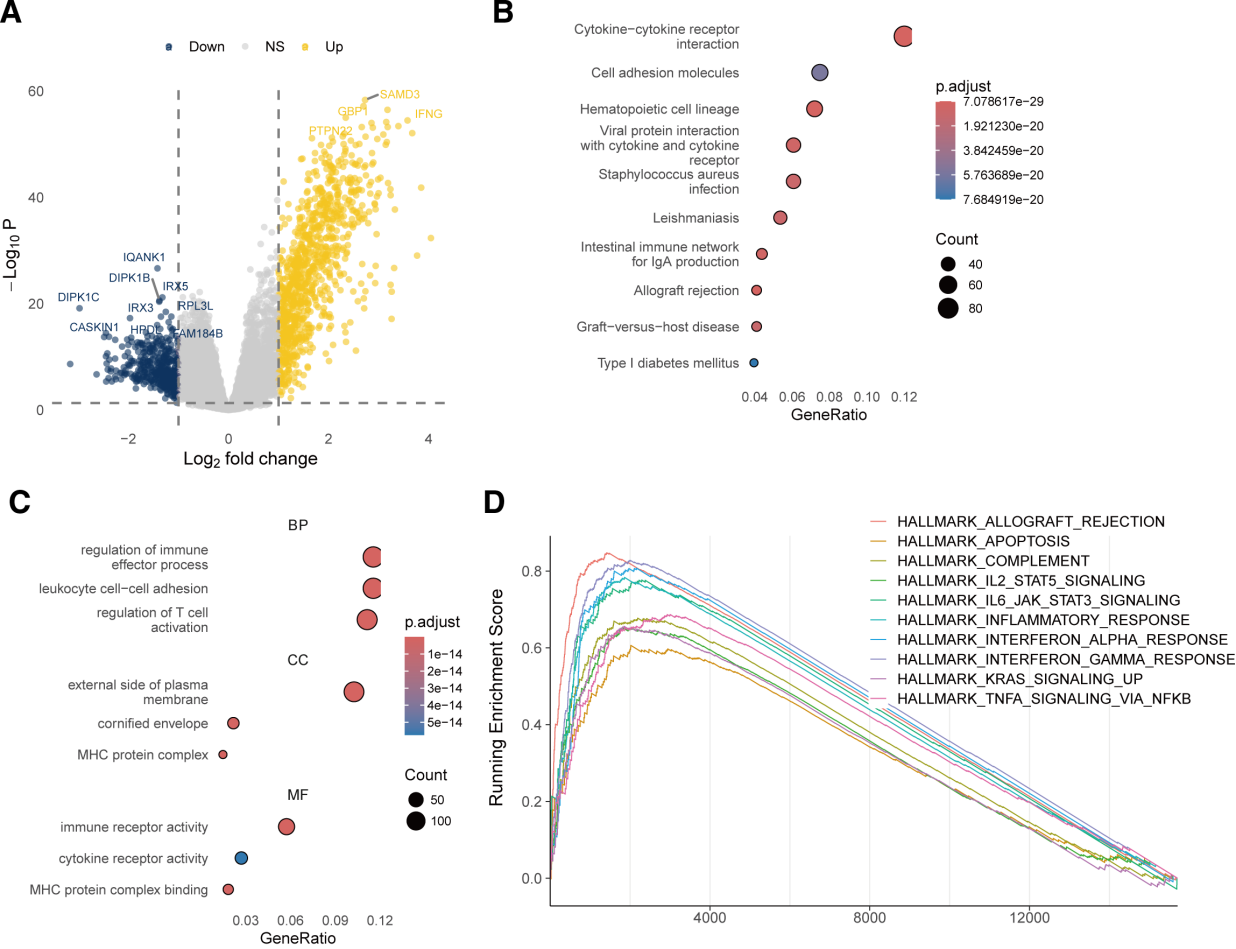
Cellular pathways and biological processes associated with VIPsig. (A) Volcano plot of differentially expressed genes (|log_2_(fold change)| > 1 and adjusted *P* < .05) between high- and low-risk groups. (B) KEGG pathway and (C) GO term enrichment analyses of VIPsig-related differentially expressed genes. (D) Gene Set Enrichment Analysis (GSEA) of Hallmark pathways. GSEA = Gene Set Enrichment Analysis, GO = Gene Ontology, KEGG = Kyoto Encyclopedia of Genes and Genomes, VIP = vasoactive intestinal peptide.

To elucidate the biological processes and pathways associated with VIPsig, we performed differential gene expression analysis between high- and low-risk groups, identifying 1338 DEGs (Fig. 6A). Enrichment analysis revealed that these genes were involved in KEGG pathways such as cytokine-cytokine receptor interaction, cell adhesion molecules, and intestinal immune network for IgA production (Fig. 6B). GO enrichment indicated associations with biological processes including immune effector process, leukocyte cell–cell adhesion, and regulation of T-cell activation (Fig. 6C). Hallmark Gene Set Enrichment Analysis further demonstrated significant activation of apoptosis, IL2-STAT5 signaling, IL6-JAK-STAT3 signaling, inflammatory response, and interferon-related pathways in the high-risk group (Fig. 6D).

### 3.7. VIPsig and tumor mutational burden in melanoma

**Figure 7.**
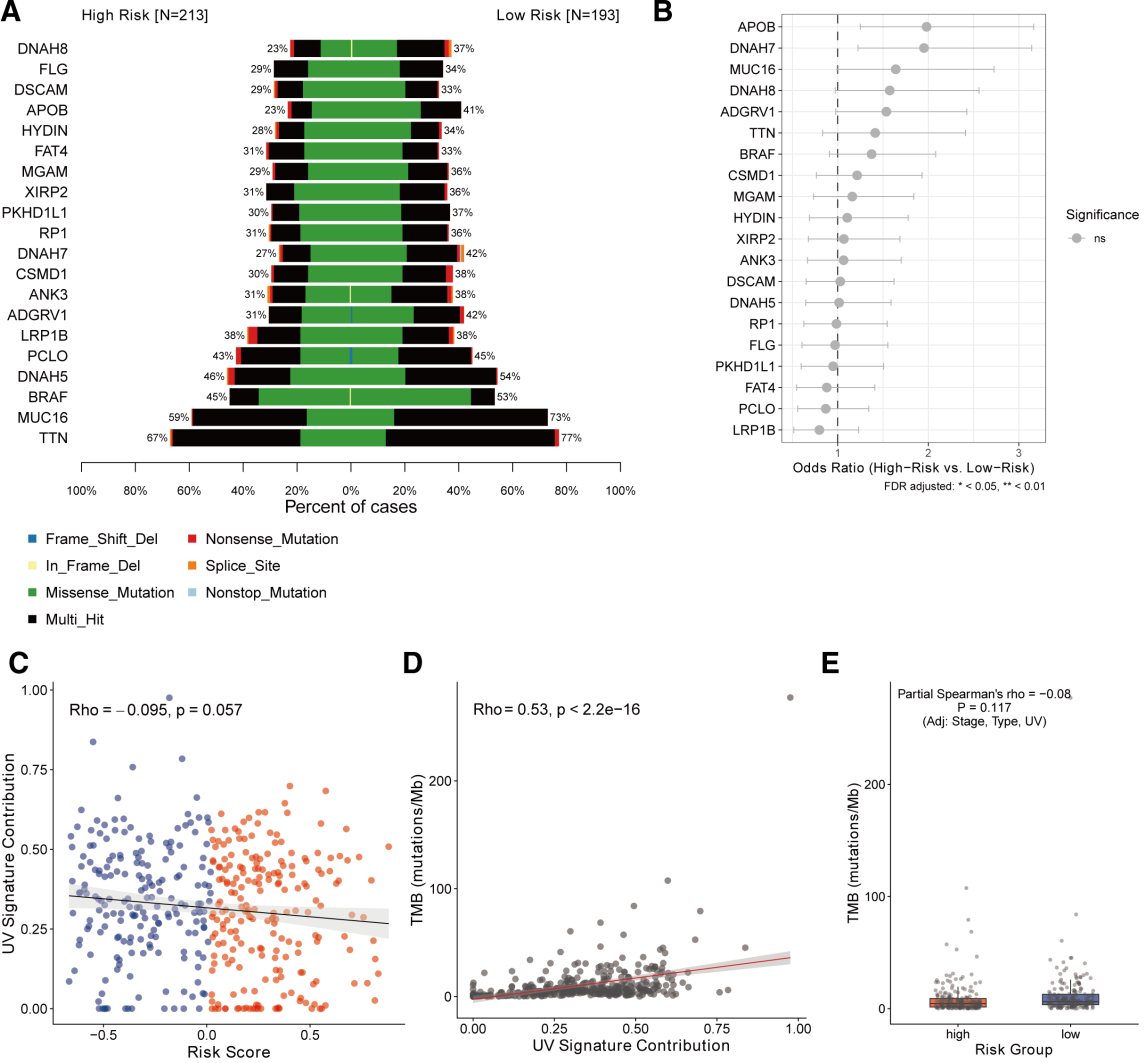
Genomic landscape and multivariable analysis of the VIPsig risk signature. (A) Co-oncoplot comparing the somatic mutation landscape of the top 20 most frequently mutated genes between the high-risk (n = 213) and low-risk (n = 193) groups. Mutation types are color-coded. (B) Forest plot displaying the odds ratios (OR) for the mutation frequencies of the top genes comparing high- versus low-risk groups. Statistical significance was assessed using Fisher’s exact test with FDR correction; “ns” indicates no significant difference (*P* > .05). (C) Scatter plot showing the correlation between the VIPsig risk score and the UV signature contribution (COSMIC Signature 7). The correlation was assessed using Spearman’s rank correlation test. (D) Scatter plot illustrating the positive correlation between tumor mutational burden (TMB) and UV signature contribution. (E) Boxplot comparing TMB levels between high- and low-risk groups. FDR = false discovery rate, OR = odds ratio, TMB = tumor mutational burden, VIP = vasoactive intestinal peptide.

We visualized the mutation profiles of the top frequently mutated genes – such as *TTN*, *MUC16*, *DNAH5*, *BRAF*, and *PCLO –* in the high- and low-risk groups (Fig. 7A). To rigorously assess whether specific mutations were enriched in either risk group, we calculated odds ratios with FDR adjustment. As shown in Figure 7B, no individual genes showed statistically significant differences in mutation frequency between the risk groups (FDR > 0.05), suggesting that the prognostic value of VIPsig is not driven by specific driver mutations. Furthermore, we investigated the relationship between VIPsig and genomic instability metrics while accounting for confounding factors. The risk score showed a marginal, nonsignificant negative trend with the UV signature contribution (ρ = −0.095, *P* = .057; Figure 7C), whereas TMB was strongly correlated with UV exposure as expected (Fig. 7D). Crucially, while TMB appeared slightly lower in the high-risk group, partial correlation analysis adjusting for tumor stage, sample type, and UV signature revealed no significant independent association between VIPsig and TMB (Partial ρ = −0.08, *P* = .117; Fig. 7E). These findings indicate that the prognostic capacity of VIPsig is independent of tumor mutational burden and UV exposure history.

### 3.8. Nomogram for melanoma patients

**Figure 8.**
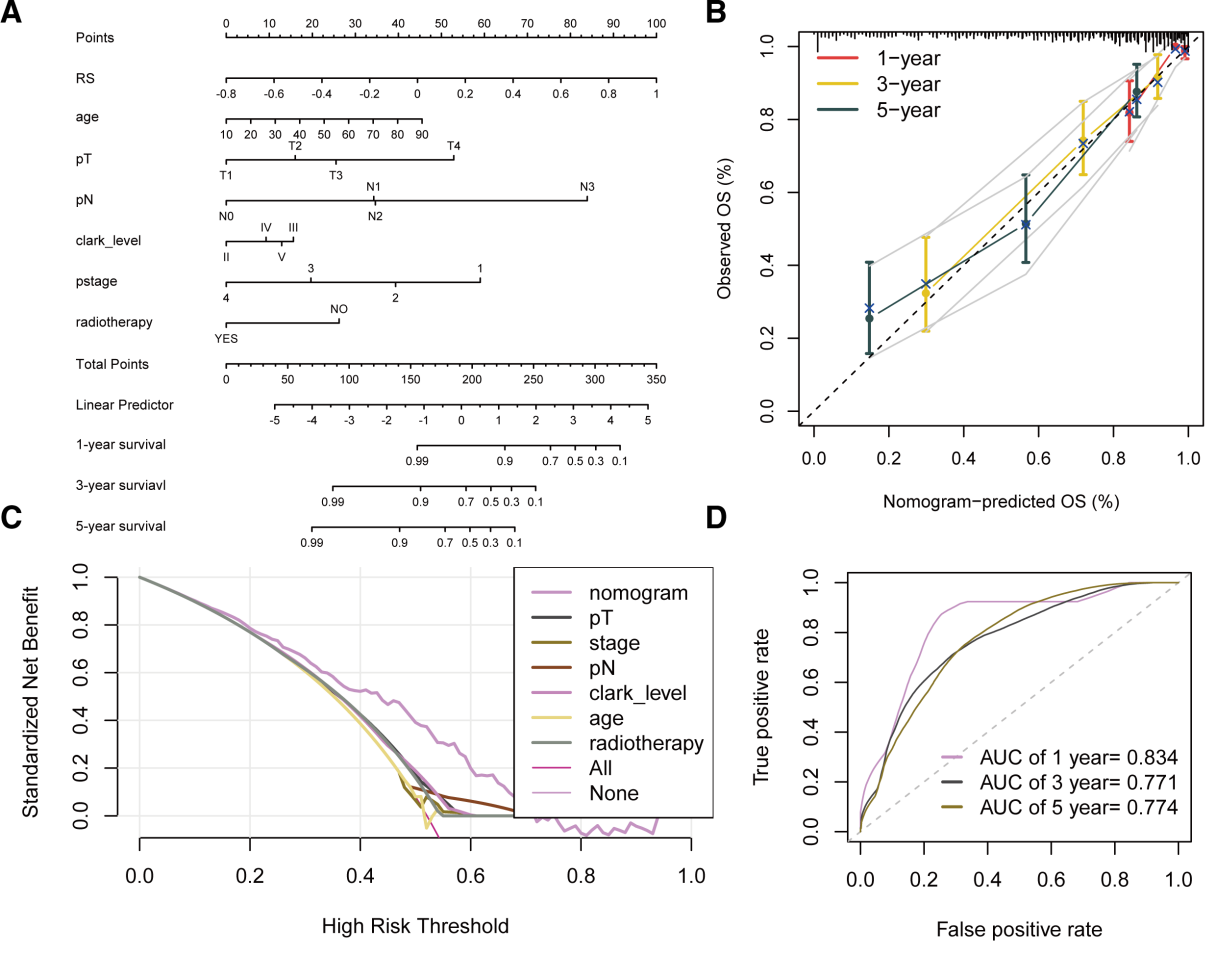
Nomogram for melanoma patients based on VIPsig and other clinicopathological features. (A) Nomogram incorporating independent prognostic factors identified by multivariate Cox analysis: VIPsig, age, pT, pN, Clark level, pStage, and radiotherapy. (B) Calibration curves for 1-, 3-, and 5-year overall survival predictions. (C) Decision curve analysis comparing the nomogram with other prognostic factors for 1-year overall survival prediction. (D) ROC curves of the nomogram for predicting 1-, 3-, and 5-year overall survival. ROC = receiver operating characteristic, VIP = vasoactive intestinal peptide.

To construct a nomogram, we first performed Cox analysis. Univariate Cox analysis identified VIPsig, age, pT, pN, pStage, Clark level, and radiotherapy as significantly associated with overall survival. Multivariate Cox analysis confirmed VIPsig, age, pT, pN, Clark level, pStage, and radiotherapy as independent prognostic factors (Table 1). These factors were incorporated into the nomogram (Fig. 8A). Calibration curves demonstrated close agreement between predicted and observed 1-, 3-, and 5-year survival probabilities in both training and validation sets, indicating good model calibration (Fig. 8B). Decision curve analysis showed that the nomogram provided higher net benefit across threshold probabilities of 0.2 to 0.8 compared to other prognostic features, supporting its clinical utility (Fig. 8C). ROC analysis revealed that the nomogram predicted 1-, 3-, and 5-year overall survival with AUC values of 0.834, 0.771, and 0.774, respectively, indicating strong discriminative ability (Fig. 8D).

**Table 1:** Univariate and multivariate cox regression analysis evaluated the prognostic relevance.

Characteristics	Univariate Cox		Multivariate Cox	
	HR [95% CI]	*P*-value	HR[95% CI]	*P*
VIPsig	6.503 [3.906–10.827]	6.09E−13	6.045 [3.4–10.746]	8.82E−10
Gender	0.916 [0.64–1.31]	.629	0.891 [0.607–1.309]	.558
Age	1.021 [1.01–1.033]	.000222	1.015 [1.003–1.027]	.0158
pT	1.702 [1.408–2.056]	3.70E−08	1.893 [1.439–2.49]	4.99E−06
pN	1.672 [1.419–1.969]	7.71E−10	2.301 [1.72–3.079]	1.96E−08
pstage	1.537 [1.261–1.874]	2.10E−05	4.061 [1.55–10.64]	.00434
clark_level	1.889 [1.465–2.435]	9.44E−07	0.431 [0.274–0.676]	.000254
pM	2.168 [0.953–4.931]	.0649	1.077 [0.79–1.47]	.638
Radiotherapy	0.271 [0.119–0.616]	.00183	0.387 [0.166–0.901]	.0277

Staging and grading variables were coded as ordered numeric integers in the Cox regression model: pT stage (T1 = 1, T2 = 2, T3 = 3, T4 = 4), pN stage (N0 = 0, N1 = 1, N2 = 2, N3 = 3), pM stage (M0 = 0, M1 = 1), pathologic stage (Stage I = 1, Stage II = 2, Stage III = 3, Stage IV = 4), and Clark level (Level I = 1, Level II = 2, Level III = 3, Level IV = 4, Level V = 5). Age and VIPsig risk score were modeled as continuous variables.

CI = confidence interval, HR = hazard ratio, VIPsig = vasoactive intestinal peptide-related gene signature.

### 3.9. Single-cell expression of VIPsig-related genes

**Figure 9.**
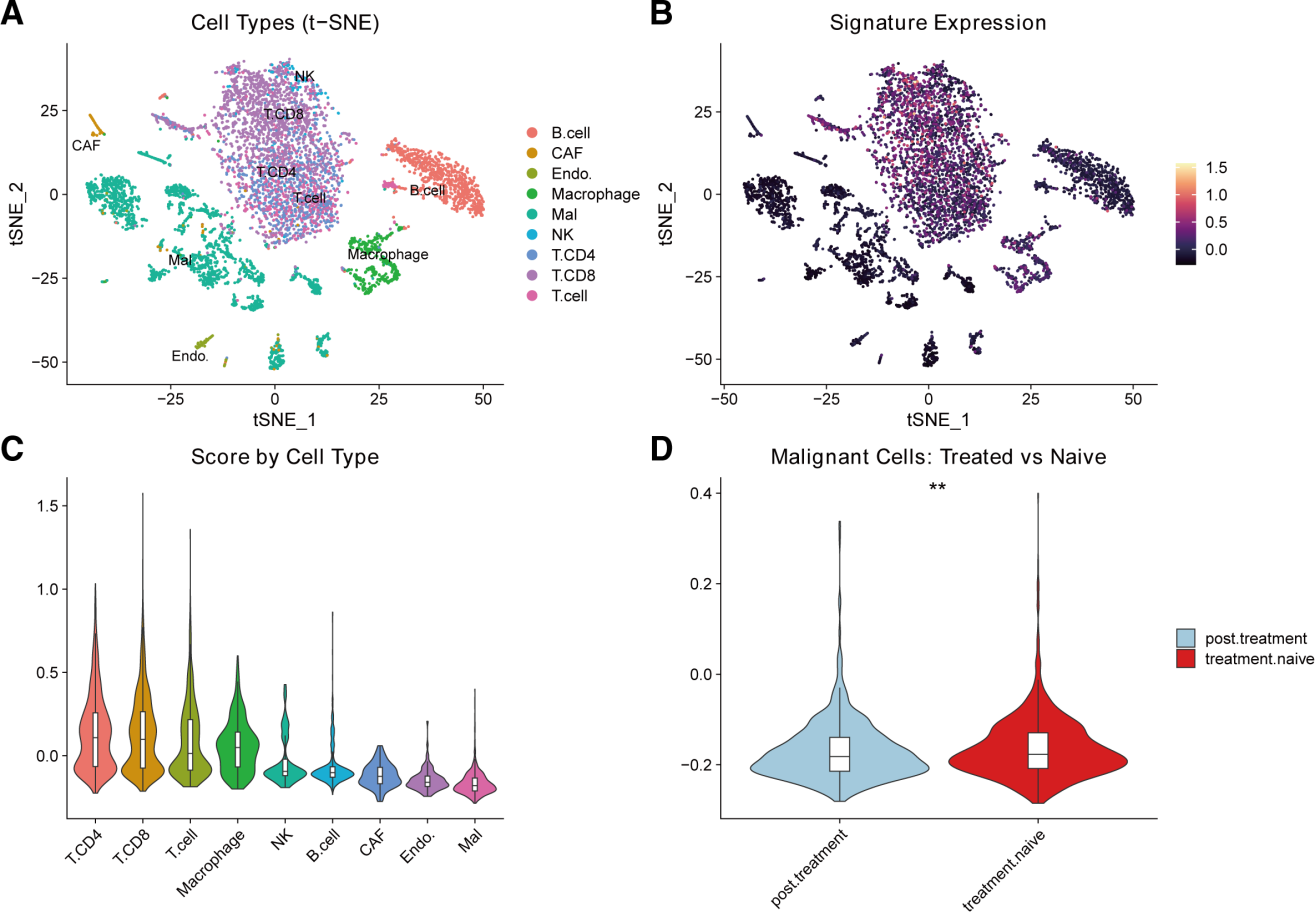
Single-Cell Transcriptomic Analysis of VIPsig in Melanoma (GSE115978). (A) t-SNE visualization of single cells from melanoma patients. Cells are colored by rigorously annotated cell types, including CD8+ T cells (T.CD8), CD4+ T cells (T.CD4), NK cells, B cells, Macrophages, Endothelial cells (Endo.), Cancer-associated fibroblasts (CAF), and Malignant melanoma cells (Mal). (B) Feature plot displaying the distribution of the VIPsig gene signature expression across the single-cell landscape. (C) Violin plot comparing the VIPsig module score across different cell types. (D) Violin plot analyzing the VIPsig score exclusively within the Malignant cell population, comparing treatment-naive versus posttreatment samples. Statistical significance was assessed using the Wilcoxon rank-sum test (** *P* < .01). CAF = Cancer-associated fibroblasts, VIP = vasoactive intestinal peptide.

To validate the cell-type specificity of VIPsig and address potential confounding by tumor purity, we reanalyzed the single-cell RNA-seq dataset (GSE115978) using expert-curated annotations. Clustering analysis identified 8 biologically distinct cell populations: malignant melanoma cells, CD8+ T cells, CD4+ T cells, NK cells, B cells, macrophages, endothelial cells, and CAFs (Fig. 9A). The accuracy of these annotations was confirmed by the expression of canonical markers, such as PMEL for malignant cells and CD3D for T cells (Fig. S3, Supplemental Digital Content), correcting previous automated labeling artifacts. Module scoring revealed that VIPsig-related genes were predominantly expressed in the immune compartment, particularly in CD8+ T cells, CD4+ T cells, and macrophages, with relatively lower expression in malignant and stromal cells (Fig. 9B, C). This observation aligns with our bulk RNA-seq findings, confirming that VIPsig largely reflects the immune infiltration status of the tumor. Crucially, to determine if differences persist after accounting for sampling heterogeneity, we analyzed the score exclusively within the malignant cell subpopulation. As shown in Figure 9D, a statistically significant difference in VIPsig scores was observed between treatment-naive and posttreatment malignant cells (*P* < .01). This suggests that while the signature is driven by immune infiltration, it also captures subtle intrinsic alterations within the tumor cells that are modulated by therapeutic pressure.

To further evaluate the clinical relevance of VIPsig in the context of immunotherapy, we analyzed the IMvigor210 cohort. Although the high-risk group showed a trend towards shorter overall survival (*P* = .093, Fig. S4A, Supplemental Digital Content) and the direct prediction of binary drug response was limited in this specific cohort (AUC = 0.543, Fig. S4B, C, Supplemental Digital Content), the biological nature of the signature was strongly validated by its association with tumor immune phenotypes. Consistent with our single-cell findings where VIPsig was associated with malignant features rather than immune effector function, the Risk Score was significantly elevated in the “immune-desert” phenotype and lowest in the “immune-inflamed” phenotype (*P* < 2.2e−16, Fig. S4D, Supplemental Digital Content). This confirms that a high VIPsig robustly characterizes an immunologically “cold” or immune-excluded tumor microenvironment, which provides a mechanistic basis for the potential resistance to immune checkpoint blockade.

## 4. Discussion

In this study, we addressed the critical challenge of stratifying melanoma prognosis by developing a novel VIP-related gene signature (VIPsig). While the immunomodulatory role of VIP is recognized in autoimmune contexts, its translational potential in oncology has remained underexplored. By integrating machine learning algorithms with transcriptomic data from TCGA-SKCM and GSE65904 cohorts, we identified a robust 8-gene signature that effectively predicts overall survival. Beyond numerical risk stratification, our multi-omics analysis – spanning bulk RNA-seq, single-cell resolution, and immunotherapy cohorts – unveiled that VIPsig serves as a surrogate marker for the tumor immune microenvironment, specifically distinguishing between immune-inflamed and immune-desert phenotypes. Compared to established signatures,^[9-11]^ VIPsig complements these by delivering stage-agnostic prognostic power (AUCs 0.76–0.77) in metastatic/mixed cohorts and by highlighting VIP-linked costimulatory inhibition and immune exclusion detectable in bulk RNA assays, which may inform future biomarker research in combination with other markers.

The 8 genes in VIPsig are predominantly immune-related, encoding molecules critical for T-cell activation, costimulation, apoptosis, and cytokine signaling. *CD28*, *CD80*, and *CD86* form the core of the costimulatory axis in T-cell priming: *CD80* and *CD86* (B7 ligands) bind *CD28* to promote effector T-cell responses, while their interaction with CTLA4 delivers inhibitory signals to maintain immune homeostasis.^[36]^ In melanoma, transfection of melanoma cells with *CD80* or *CD86* enhances antitumor immune responses by promoting T-cell proliferation, underscoring their role in bridging innate and adaptive immunity.^[37]^ Clinicopathologic studies further indicate that elevated *CD80* and *CD86* expression correlates with better prognosis in melanoma, potentially reflecting enhanced antigen presentation and immune activation – patterns mirrored in our VIPsig’s negative correlation with pro-tumor macrophages and positive association with immune checkpoints.^[38]^ *CTLA4*, a key inhibitory receptor, is upregulated by IFNG signaling in melanocytes and melanoma cells, creating a feedback loop that dampens T-cell effector function and fosters immune exclusion.^[39,40]^ This is evidenced by the efficacy of *CTLA4* blockade (e.g., ipilimumab) in melanoma, where disrupting *CTLA4*-*CD80*/*CD86* interactions reinvigorates exhausted T cells, though IL-21 and IL-17 signaling modulates this response to enhance checkpoint therapy outcomes.^[41]^ *FAS (CD95*) mediates apoptosis in activated T cells and tumor cells, linking to stromal-immune crosstalk by regulating FasL-FAS interactions in the TME, which can promote tumor cell survival if dysregulated.^[36]^ Cytokines like *IFNG* (interferon-gamma) drive pro-inflammatory Th1 responses essential for anti-melanoma immunity, while *IL12A* promotes NK and T-cell activation; conversely, IL10 acts as an immunosuppressive factor that inhibits dendritic cell maturation and fosters regulatory T cells (Tregs), contributing to immune tolerance in advanced melanoma.^[42]^ These genes collectively participate in stromal-immune crosstalk: for instance, melanoma-associated fibroblasts (CAFs) can upregulate *CTLA4* and *IL10* to exclude effector cells from the tumor core, aligning with our single-cell findings of VIPsig expression in immune compartments and its correlation with tumor purity.^[43]^ VIP may indirectly regulate this network via *VPAC* receptors on immune cells, amplifying *CTLA4*-mediated inhibition and *IL10*-driven suppression to create an immune-desert phenotype. Co-expression correlations in our data and the observed negative association with CD8+ T and NK cell infiltration support this, but direct VIP knockout studies in melanoma models are needed to confirm causality, distinguishing our correlative findings from mechanistic proof.

The immune landscape analysis robustly characterized VIPsig as a marker of an immunologically “cold” or immune-excluded TME. Cross-platform deconvolution consistently revealed that a high VIPsig score was associated with reduced infiltration of antitumor effector cells (e.g., CD8+ T cells, activated NK cells) and enriched pro-tumorigenic populations (e.g., M0/M2 macrophages). This aligns with the negative correlation between VIPsig and stromal/immune scores, and its positive correlation with tumor purity, collectively depicting a TME less conducive to immune surveillance. Notably, this suppressive net effect captured by the integrated VIPsig contrasts with the pro-inflammatory functions of its individual components (e.g., *CD28*, *CD80*/*CD86*, *IFNG*, *IL12A*), which typically promote immune activation. This paradox may be resolved by considering VIP-mediated immunoregulatory feedback.^[17]^ VIP signaling is known to induce tolerance, potentially creating a context where the co-expression of these activation-associated genes reflects a dysregulated or exhausted immune state rather than productive immunity. Our single-cell analysis corroborates this by showing that VIPsig-related gene expression is predominantly localized to the immune compartment, confirming the signature’s reflection of immune contexture. Importantly, a significant difference in the VIPsig score was also detected within the malignant cell subpopulation between treatment-naive and posttreatment conditions, suggesting it may also capture therapy-induced intrinsic alterations. The clinical relevance of this “cold” phenotype was further supported by analysis of the IMvigor210 cohort, where a high VIPsig score was significantly associated with the “immune-desert” phenotype. Thus, VIPsig appears to identify melanoma patients with a TME characterized by immune exclusion and potential diminished responsiveness to ICIs,^[44,45]^ warranting further clinical investigation.

The exploratory in silico drug sensitivity findings, while hypothesis-generating, may derive from the interplay between VIPsig genes and drug mechanisms. It is important to emphasize that these computational predictions are based on cell line-derived pharmacogenomic data and have not been validated in clinical melanoma samples or prospective trials. For instance, IFNG and IL12A, which promote inflammatory signaling, could enhance responses to immunomodulatory drugs like lenalidomide, which stimulates cytokine production and NK cell activity.^[46,47]^ Conversely, the negative correlation with imatinib might relate to CTLA4 and CD28’s roles in T-cell signaling, potentially modulating tyrosine kinase pathways in the TME.^[48-50]^ FAS-mediated apoptosis could influence sensitivity to DNA-damaging agents like gemcitabine,^[51]^ where high VIPsig (potentially reduced FAS activity) might confer resistance. The immunosuppressive TME in high-VIPsig tumors could also indirectly affect drug efficacy by altering drug delivery or immune-mediated clearance. It is important to note that the initial inverse trend observed between VIPsig and TMB was not statistically significant after adjusting for key covariates including tumor stage and UV exposure (Partial ρ = −0.08, *P* = .117). Thus, the drug sensitivity patterns predicted for high-VIPsig tumors are likely mediated by mechanisms other than mutational load, such as the specific immune-excluded phenotype this signature captures.

Differential expression analysis between high- and low-VIPsig groups identified 1338 genes, with enrichment analyses illuminating underlying biological processes. KEGG pathway enrichment pointed to cytokine-cytokine receptor interaction, cell adhesion molecules, and intestinal immune network for IgA production, emphasizing immune regulation. GO terms highlighted immune effector processes, leukocyte cell-cell adhesion, and regulation of T-cell activation, reinforcing VIPsig’s immunomodulatory role. Hallmark gene set enrichment revealed activation of apoptosis, IL2-STAT5 signaling, IL6-JAK-STAT3 signaling, inflammatory response, and interferon-related pathways in the high-VIPsig group. These enrichments tie directly to VIPsig genes: *IL10* and *IL12A* are central to cytokine networks, modulating inflammatory responses^[52,53]^; *IFNG* drives interferon pathways^[54]^; and *CTLA4*/*CD28* regulate T-cell activation via STAT signaling.^[49,55]^ The activation of apoptosis and inflammatory pathways in high-VIPsig tumors might represent a compensatory response to immune suppression, or alternatively, a tumor-promoting mechanism via chronic inflammation. This duality mirrors VIP’s pleiotropic effects, where it can suppress acute inflammation but sustain low-grade chronic states favorable for tumor growth.

In acknowledging the potential limitations of the study, we recognize several key areas for improvement. First, the retrospective design limits causal inference; while VIPsig’s associations with TME and survival are consistent across cohorts, prospective clinical trials are essential to assess its utility in real-time decision-making. Second, although we adjusted for confounders like stage and purity, unmeasured variables (e.g., microbiome influences on IL10 signaling) could bias results. Third, the focus on bulk and single-cell RNA-seq overlooks posttranscriptional regulation (e.g., miRNA modulation of CTLA4), and the IMvigor210 validation showed only marginal OS differences (*P* = .093), highlighting cohort-specific variability. Furthermore, it should be noted that the joint application of ComBat for batch-effect correction across the combined cohorts may have allowed some distributional information from the validation set into the preprocessing stage, which could potentially result in a slight overestimation of the signature’s performance in external validation. Fourth, in silico analyses, while computationally efficient, cannot fully replicate clinical responses; experimental validation of VIP-VPAC targeting in VIPsig-stratified models is crucial. Finally, generalizability to non-Caucasian populations or uveal melanoma remains untested, as TCGA is predominantly skin melanoma. Critically, while VIPsig demonstrated strong prognostic associations, its predictive utility for treatment response – particularly to ICIs – remains unproven. The IMvigor210 cohort analysis showed only marginal survival differences (*P* = .093) and limited binary response discrimination (AUC = 0.543), highlighting that translation to therapeutic decision-making requires validation in melanoma-specific immunotherapy cohorts. Future studies should integrate proteomics and spatial transcriptomics to dissect VIP-driven stromal-immune interactions more granularly.

## 5. Conclusion

In conclusion, VIPsig represents a novel prognostic biomarker in melanoma that is closely associated with immune suppression and inflammatory pathway activity. The exploratory in silico analyses suggest potential associations with drug sensitivity estimates, but these findings are hypothesis-generating and do not constitute validated treatment response predictions. Notably, although VIPsig was associated with immune phenotypes in the IMvigor210 cohort, its direct predictive value for immunotherapy response was limited (AUC = 0.543), underscoring the need for validation in melanoma-specific cohorts treated with ICIs. By bridging bulk and single-cell insights, this signature offers a framework for future mechanistic studies to unravel VIP’s roles in tumor immunity and to prospectively evaluate its clinical utility.

## Author contributions

**Conceptualization:** Xiaogang Hong, Xiuzhen Zheng.

**Data curation:** Xiaogang Hong, Xiuzhen Zheng.

**Formal analysis:** Xiaogang Hong, Xiuzhen Zheng.

**Funding acquisition:** Xiaogang Hong, Xiuzhen Zheng.

**Investigation:** Xiaogang Hong, Xiuzhen Zheng.

**Methodology:** Xiaogang Hong, Xiuzhen Zheng.

**Project administration:** Xiaogang Hong, Xiuzhen Zheng.

**Resources:** Xiaogang Hong, Xiuzhen Zheng.

**Software:** Xiaogang Hong, Xiuzhen Zheng.

**Supervision:** Xiaogang Hong, Xiuzhen Zheng.

**Validation:** Xiaogang Hong, Xiuzhen Zheng.

**Visualization:** Xiaogang Hong, Xiuzhen Zheng.

**Writing – original draft:** Xiaogang Hong, Xiuzhen Zheng.

**Writing – review & editing:** Xiaogang Hong, Xiuzhen Zheng.



**Figure:**
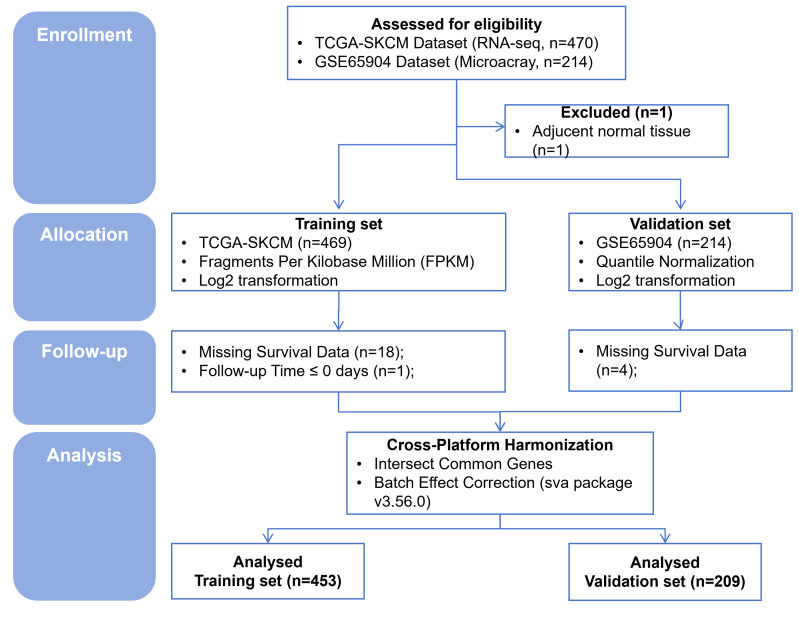




**Figure:**
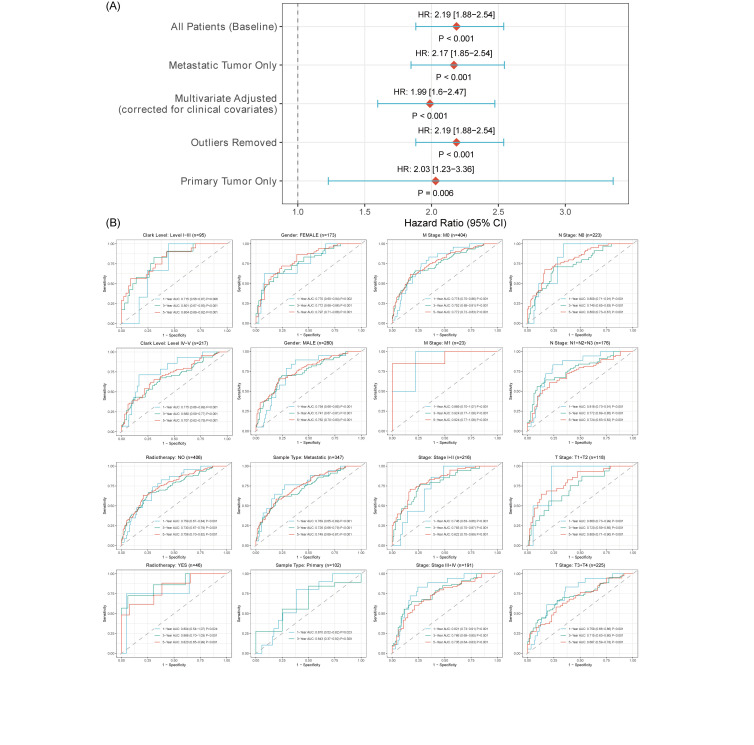


**Figure:**
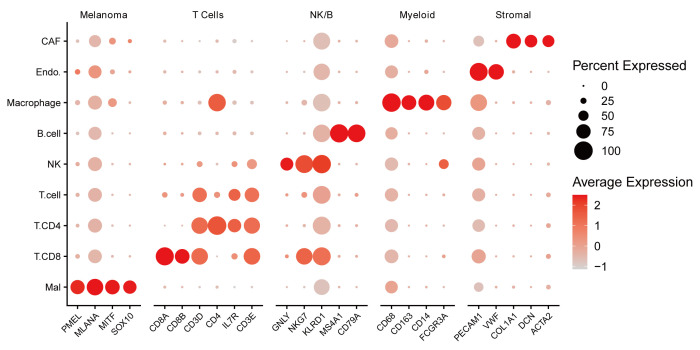


**Figure:**